# Process optimization for gold nanoparticles biosynthesis by *Streptomyces albogriseolus* using artificial neural network, characterization and antitumor activities

**DOI:** 10.1038/s41598-024-54698-2

**Published:** 2024-02-25

**Authors:** Noura El-Ahmady El-Naggar, Asmaa A. El-Sawah, Mohamed F. Elmansy, Omar T. Elmessiry, Mohanad E. El-Saidy, Mostafa K. El-Sherbeny, Mohamed T. Sarhan, Aya Amin Elhefnawy, Shimaa R. Dalal

**Affiliations:** 1 Department of Bioprocess Development, Genetic Engineering and Biotechnology Research Institute, City of Scientific Research and Technological Applications (SRTA-City), New Borg El- Arab City, Alexandria, 21934 Egypt; 2 Botany Department, Faculty of Science, Mansoura University, Mansoura, 35516 Egypt; 3 Biotechnology and its Application Program, Department of Botany, Faculty of Science, Mansoura University, Mansoura, 35516 Egypt

**Keywords:** GNPs, *Streptomyces albogriseolus*, Biosynthesis, Characterization, Process optimization, Central composite design, Artificial neural network, HeP-G2 human cancer cell line, Nanoparticles, Applied microbiology

## Abstract

Gold nanoparticles (GNPs) are highly promising in cancer therapy, wound healing, drug delivery, biosensing, and biomedical imaging. Furthermore, GNPs have anti-inflammatory, anti-angiogenic, antioxidants, anti-proliferative and anti-diabetic effects. The present study presents an eco-friendly approach for GNPs biosynthesis using the cell-free supernatant of *Streptomyces albogriseolus* as a reducing and stabilizing agent. The biosynthesized GNPs have a maximum absorption peak at 540 nm. The TEM images showed that GNPs ranged in size from 5.42 to 13.34 nm and had a spherical shape. GNPs have a negatively charged surface with a Zeta potential of − 24.8 mV. FTIR analysis identified several functional groups including C–H, –OH, C–N, amines and amide groups. The crystalline structure of GNPs was verified by X-ray diffraction and the well-defined and distinct diffraction rings observed by the selected area electron diffraction analysis. To optimize the biosynthesis of GNPs using the cell-free supernatant of *S. albogriseolus*, 30 experimental runs were conducted using central composite design (CCD). The artificial neural network (ANN) was employed to analyze, validate, and predict GNPs biosynthesis compared to CCD. The maximum experimental yield of GNPs (778.74 μg/mL) was obtained with a cell-free supernatant concentration of 70%, a HAuCl_4_ concentration of 800 μg/mL, an initial pH of 7, and a 96-h incubation time. The theoretically predicted yields of GNPs by CCD and ANN were 809.89 and 777.32 μg/mL, respectively, which indicates that ANN has stronger prediction potential compared to the CCD. The anticancer activity of GNPs was compared to that of doxorubicin (Dox) in vitro against the HeP-G2 human cancer cell line. The IC_50_ values of Dox and GNPs-based treatments were 7.26 ± 0.4 and 22.13 ± 1.3 µg/mL, respectively. Interestingly, treatments combining Dox and GNPs together showed an IC_50_ value of 3.52 ± 0.1 µg/mL, indicating that they targeted cancer cells more efficiently.

## Introduction

Nanotechnology is a multidisciplinary field concerned with the fabrication, characterization, and applications of nanoparticles (NPs) ranging from 1 to 100 nm. The tremendous interest in the metal-NPs synthesis is due to their remarkable capabilities as catalysts and assistance in various processes in biology, medicine, physics, engineering, chemistry, and informatics^1^. NPs have distinct physicochemical characteristics in comparison to their solid bulk materials, owing to their shape, size, greater surface area-to-volume ratio, chemical, optical and electronic properties^2^.

GNPs have attracted a lot of interest among various metal-NPs due to their special optical, catalytic, and biomedical characteristics as well as their non-toxicity and biocompatibility^3^. The surface of GNPs can be easily functionalized and bio-conjugates by several biological compounds such as peptides, proteins, amino acids, oligonucleotides, therapeutic and/or diagnostic agents, DNA/RNA, antibodies and/or tumour markers to specifically target receptors or cell surface proteins on cancer cells^4,5^. The binding of these biomolecules with GNPs confer specificity for cellular targets making GNPs a promising candidate for clinical applications in targeted drug and bio-macromolecule delivery^6^.

The safety or toxicity of green synthesized metal-NPs in relation to cancerous and normal cell lines is a substantial concern. Metal-NPs may be used as anti-cancer drugs if their cytotoxicity against cancerous cell lines much more than toward normal cell lines^4^. A great advantage of GNPs over other NPs is that they show no cytotoxicity in human cells. A series of GNPs were examined for uptake and acute toxicity in human leukemia cells. The results indicate that the spherical GNPs with a variety of surface modifiers are not inherently toxic to human cells, despite being taken up into cells and not detrimental to cellular function^7^. GNPs have been utilized successfully in a wide range of biomedical applications, particularly in therapy and diagnostics, such as cancer treatment, photothermal therapy, vaccine development, the treatment of rheumatoid arthritis, as well as antiviral and antibacterial drugs^8^. In addition, GNPs are used in drug and gene delivery, surgery and medicine, bioimaging and biosensors. GNPs display plasmonic sensing in the presence of surface-binding biomolecules. Therefore, GNPs have emerged as the optimal choice for biosensing because of its exceptional optical characteristics, chemical stability, and ease of bioconjugation. The aggregation of spherical gold plasmonic NPs in a colloidal solution in the presence of molecules as DNA leads to the solution changes colour from red to blue^9^. Tomar and Garg^10^ demonstrated that GNPs aggregate and scatter light in tumor cells. Therefore, GNPs can serve as a probe for the microscopic examination of cancer cells. Because of their optical characteristics, GNPs are used as contrast agents in X-ray and optical imaging technologies, which enable the detection of fibrotic tissue, intravascular thrombus, and atherosclerotic plaques^11^. GNPs have the potential to serve as promising therapeutic agents for the treatment of diabetes and its associated microvascular complications. This ability originates from their anti-fibrotic, anti-hyperglycemia, anti-oxidation, anti-inflammation, anti-glycation, diabetic wound healing, and anti-angiogenic capabilities^12^. Ranjitha and Rai^3^ reported that, GNPs exhibited strong catalytic activity for the methylene blue degradation.

NPs have traditionally been synthesised using a variety of chemical and physical techniques. The main drawbacks of chemical techniques are the use of very toxic chemicals, carcinogenic solvents, and contaminated precursors. Physical techniques such as laser ablation require expensive equipment and use a lot of energy. Chemical and physical techniques are less effective due to NPs instability, challenges in controlling the particles formation process, and the tendency of particles to agglomerate. The green method of producing NPs is currently receiving more attention as a great alternative to conventional chemical and physical methods due to the use of non-toxic natural renewable resources, low energy consumption, and being less expensive and eco-friendly^13,14^.

Microorganisms were used for green synthesis of NPs. Actinomycetes including *Streptomyces* sp. are commonly employed in the synthesis of NPs due to their capacity to produce diverse bioactive secondary metabolites and extracellular enzymes^15–19^. Additionally, algal pigments^1,2^, algal derived soluble polysaccharides^20^, fungi^21^ and plant leaves extracts^22–24^ were used for the biosynthesis of NPs due to their favorable impact on both the synthesis process and the final NPs characteristics. Microbial metabolites and phytochemicals can act as bio-reducers and/ or stabilizing/capping agents in the biosynthesis process^14,23^. Microbial biosynthesis is viewed as a great alternative method for the extracellular synthesis of GNPs^25^. Many types of proteins can participate in the reduction of chloroauric ions; have the ability to function as capping agents and stabilizing the surfaces of metal-NPs^26^.

The optimization of medium components is a vital step in the synthesis of GNPs. The initial optimization technique, known as the factor-by-factor technique or traditional technique, involves changing one of the independent variables while keeping the other variables at their optimal levels. The factor-by-factor technique has some disadvantages, such as being tedious, laborious, time-consuming, and costly. Furthermore, the interaction effects between the independent variables are ignored by the traditional technique. One statistical and mathematical technique that can be used to optimise multiple variables at once is central composite design (CCD). Compared to traditional methods, CCD is cost effective, faster, reduces the total number of the experimental trials, defines the optimal process conditions, and maintains a high degree of accuracy in the final result. In biotechnology, artificial neural networks (ANNs) are the most used artificial learning method. ANNs can be used for many applications, including the bioprocesses optimization^23^.

Metal-NPs induced cytotoxicity responses are influenced by various parameters including size distribution, shape, surface charge, capping agent, and surface area^5^. Ahmed et al.^27^ reported that most of the biosynthesized GNPs using *Rhodopseudomonas capsulate* sizes ranged from 10 and 20 nm. Shakibaie et al.^28^ used the cell extract of microalga *Tetraselmis suecica* as a reducing agent to synthesize 79 nm spherical GNPs. Safarpoor et al.^29^ reported that the plant *Linum usitatissimum* plant can produce GNPs ranging from 20 to 60 nm. According to the findings of Bhat et al.^30^, the edible mushroom *Pleurotus florida* is able to produce GNPs with a varying size of 10–50 nm. Our findings demonstrated that the biosynthesized GNPs using the cell-free supernatant of *S. albogriseolus* ranged in size from 5.42 to 13.34 nm, indicating that the biosynthesis approach adopted in this study is advantageous in terms of GNPs size. Gold nanorods were discovered to be more cytotoxic than gold nanospheres^31^.

This study aims to use a biological technique to synthesize GNPs using the cell-free supernatant of *S. albogriseolus*, to optimize the biosynthesis process of GNPs and verify their synthesis using UV–vis spectrophotometric analysis, to characterize the biosynthesized GNPs using TEM, FTIR spectroscopy, XRD, EDX and to evaluate GNPs for its potential for cancer treatment. To the best of our knowledge, this is the first report that highlights the effective use of *S. albogriseolus*'s cell-free supernatant as an innovative tool for GNPs biosynthesis, as well as an artificial intelligence-based optimization approach for GNPs biosynthesis.

## Materials and methods

### Microorganism’s cultural conditions

The first author kindly provided the *Streptomyces* strain used in this study. *S. albogriseolus* was cultured on plate cultures that contained starch nitrate agar medium of the following components (g/L): Starch 20; CaCO_3_ 3; K_2_HPO_4_ 1; KNO_3_ 2; MgSO_4_.7H_2_O 0.5; NaCl 0.5; FeSO_4_.7H_2_O 0.01; distilled water up to one liter and agar 20 g/L. The Petri plates were incubated at 30 °C for seven days. The suspension of *S. albogriseolus* spores was preserved at − 20 °C a 20% glycerol solution.

### Inoculum preparation

*S. albogriseolus* was grown on starch nitrate agar plates for seven days at 30 °C. Medium consisted of the following components (g/L): yeast extract 0.3; soluble starch 20; KNO_3_ 1; MgSO_4_.7H_2_O 0.5; NaCl 0.5; K_2_HPO_4_ 0.5; FeSO_4_.7H_2_O 0.01 in addition to 1 L of distilled water, pH adjusted to 7–7.5 was prepared. Erlenmeyer flasks with a capacity of 250 mL containing 50 mL of the previously prepared medium were sterilized before being inoculated with three 9 mm diameter culture discs. The inoculated flasks were incubated for 5 days at 30 °C and 150 rpm in a shaker incubator. The obtained cultures served as an inoculum in subsequent experiments.

### Extracellular biosynthesis of GNPs

The cells-free supernatant of *S. albogriseolus* was used to synthesize GNPs. A stock solution of 1000 μg/mL gold (III) was prepared by dissolving precise amount of Gold (III) chloride trihydrate (HAuCl_4_.3H_2_O) in distilled water. The cells-free supernatant of *S. albogriseolus* was mixed with varying concentrations of HAuCl_4_ solution at a 1:2 volume-to-volume ratio, and the mixture was then incubated at 37 °C in the dark for 24–96 h. After the incubation period, the mixture's color changed to red or dark purple as a result of the reduction of gold chloride and GNPs formation. As a control, the cells-free supernatant was used without the addition of HAuCl_4_ solution.

### Characterization of GNPs using UV–visible spectrophotometer

For confirmation of the GNPs biosynthesis and HAuCl_4_ reduction, A UV–visible spectrophotometer (Optizen Pop) was used to scan the reaction mixture. After incubation, the reaction mixture was scanned using UV–Vis spectroscopy to detect the maximum absorption between 300 and 800 nm. The slit width of the measurement was 10 nm. The cells-free supernatant was used without the addition of HAuCl_4_ solution as a control.

### Microscopy analysis of GNPs

Transmission electron microscopy (TEM; JEOL-JEM-2100 Plus, Ltd., Japan) was used to determine the elemental composition of the sample by EDX analysis, to examine the structure, size, and morphological properties of GNPs samples. Also, TEM was used for mapping analysis and to analyze the selected area electron diffraction (SAED). In order to prepare the GNPs suspension for analysis, it was first submerged in an ultrasonic bath for 10 min. About 10 μL of the sample was applied to a carbon-coated grid of copper for about 30 s. The excess liquid was removed using filter paper, and the grids were then left to dry.

### Zeta potential analysis of GNPs

The suspension stability and surface charge of GNPs can be determined with the use of the zeta potential analysis. The Malvern 3000 analytical Zeta sizer Nano ZS instrument from UK; with a laser Doppler was used to measure the zeta potential and surface charge characteristics of GNPs. Deionized water was used to dilute the NPs in order to reduce the number of scattering effects that were occurring. After dissolving the sample, the NPs were quantified over a calibrated area measuring 2 mm at a count rate of 101.9 kilo counts per second (kcps) for a duration of 60 s.

### FTIR analysis of GNPs

FTIR spectroscopy was used to examine the surface properties of GNPs and characterize their chemical structure. To examine the surface characteristics, GNPs specimen has been mixed and crushed with KBr pellets. The FTIR spectrum of GNPs was measured using a Shimadzu FTIR-8400 S spectrophotometer, with a resolution of about 1 cm^−1^ and the spectrum was determined between 4500 and 500 wave number (cm^−1^).

### X-ray diffraction analysis of GNPs

The structural characteristics and crystalline nature of GNPs were investigated using XRD (Bruker D2 Phaser 2nd Gen) which was fitted with a CuKα radiation, λ = 1.5406 A°. The applied voltage used was 10 mA and 30 kV. Diffraction intensity was measured at a scanning speed of 2°/min, and the measured values of 2*θ* ranged between 0 and 80°.

### Central composite design (CCD) for GNPs optimization

The optimum value for each of the four independent factors and their effects on the biosynthesis of GNPs were determined using central composite design (CCD). The four examined independent variables were HAuCl_4_ concentrations (200–1000 µg/mL), initial level of pH (6–10), CFS concentration (60–100%) and incubation time (24–120 h). Each of the four variables varies at five different levels. A total of thirty experimental trials were carried out, from which six runs were carried out at the central levels. The correlations between the process independent variables and the biosynthesis of GNPs could be calculated using the second-order polynomial equation, providing a response value (µg/mL).

1 $$Y = \beta_{0} + \sum\limits_{i} {\beta_{i} X_{i} + \sum\limits_{ii} {\beta_{ii} } } X_{i}^{2} + \sum\limits_{ij} {\beta_{ij} } X_{i} X_{j}$$

In which Y is the predicted biosynthesis of GNPs (µg/mL), the coded values of the independent variables are represented by *X*_*i*_, the regression coefficient is represented by *β*_0_, the linear coefficient is represented by *β*_*i*_, the quadratic coefficients are represented by *β*_*ii*_ while the interaction coefficients are represented by *β*_*ij*_.

### Artificial neural network (ANN) analysis

The ANN analysis, conducted through JMP Pro 14 Software. To perform an ANN analysis, the matrix and the experimental findings of CCD were used. To assess the prediction potential of ANN analysis, the CCD data was divided into training and validation testing sets. The ANN architecture is made up of 20 hidden layers that were analyzed based on a number of factors, such as learning rates, holdback propagation ratios, and the number of neurons. The input layer contains the four independent factors including HAuCl_4_ concentrations, initial level of pH, CFS concentration and incubation time (four neurons), while the output layer contains only one neuron (GNPs biosynthesis by the cells-free supernatant of *S. albogriseolus*, µg/mL). To evaluate the ANN predictive efficacy compared to CCD, the trial-and-error method was employed. A comparison was made between the CCD and ANN models to determine the best model predict GNPs biosynthesis very near to the experimental values of GNPs biosynthesis. The model efficacy was determined based on R^2^ values, SSE, MAD, and RMSE.

### Statistical analysis

The experimental design and statistical analysis were carried out using the Windows software Design Expert version 12 (https://www.statease.com/software/design-expert/). Using the STATISTICA programme (Version 8.0, StatSoft Inc., Tulsa, USA), three-dimensional surface plots were constructed (https://www.statsoft.de/de/software/statistica). JMP pro 14 software was used to perform the artificial neural network (ANN) analysis (https://www.jmp.com/en_in/home.html).

### Antitumor activity of GNPs

#### Cell culture

The hepatic cancer cell line (HeP-G2, ATCC number HB-8065) was obtained from the Holding Company for Biological Products and Vaccines (VACSERA), Cairo, Egypt. The HeP-G2 was cultured in RPMI-1640 media with 10% fetal bovine serum (FBS) and 1% (v/v) antibiotics (100 µg/mL streptomycin and 100 U/mL penicillin), then maintained at 37 °C in a CO_2_ (5%) incubator (Binder, C-series, Germany).

#### Cytotoxic assay

The in vitro antitumor activity of GNPs against HeP-G2 was evaluated using an MTT assay^32^ based on the cell metabolic activity to reduce the tetrazolium salt. In brief, 10 × 10^3^ cells/well were seeded in 96-well plates (Griener, Germany). The cells were then incubated for 24 h at 37 °C with 5% CO_2_, 100% relative humidity, and 95% air to help the cells adhere to the bottoms of the wells. Fresh serum-free media containing DMSO at a final concentration of 0.1% was substituted for the old medium. At first, the GNPs were passed through a 0.45-m filter syringe, then employed to treat the cells at different concentrations (1.56, 3.125, 6.52, 12.5, 25, 50, and 100 µg/mL) for another 24 h with 5% CO_2_ and 100% relative humidity at 37 °C. Doxorubicin (DOX) was utilized as the commercial reference. In each well, 5 mg/mL of PBS and 20 µL of the yellow MTT solution were added for 4 h at 37 °C for MTT reduction. The resulting purple formazan product was mixed with 100 µL of DMSO, and an EXL 800 plate reader was used to measure the absorbance at 570 nm. The viability percentage has been calculated by:

2 $${\text{Viability}}\% = \left( {{\text{Test}}\;{\text{OD}}/{\text{Control}}\;{\text{OD}}} \right) \times {1}00$$

## Results and discussion

### Extracellular biosynthesis of GNPs

**Figure 1:**
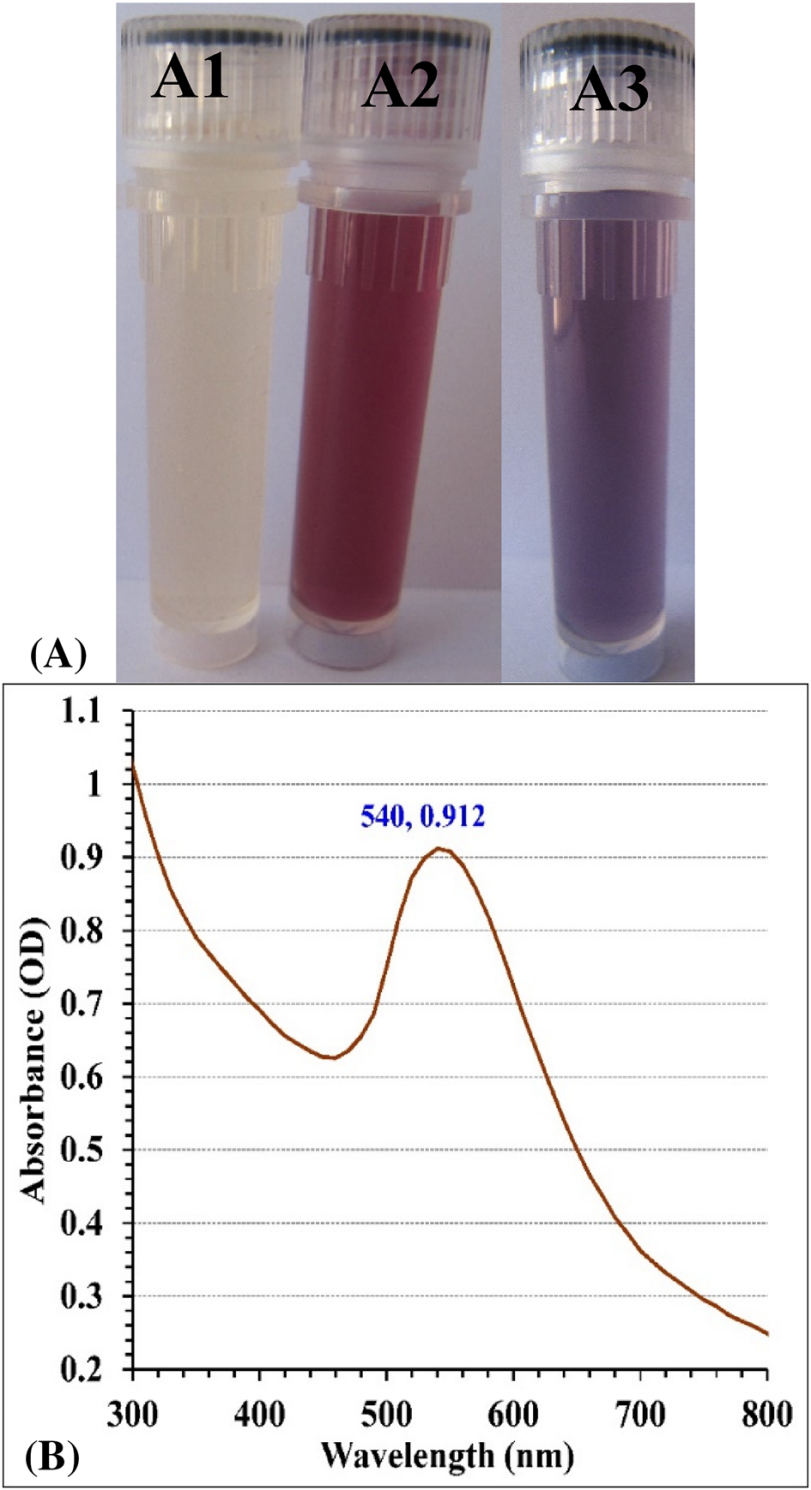
(**A**) Vials of: A1 cell-free supernatant, A2 GNPs solution after 24 h and A3 GNPs solution after 96 h of incubations; (**B**) UV.vis absorbance of GNPs (Tube A2l; a single SPR band at 540 nm).

This study presents an eco-friendly, cost-effective, and biosafe protocol for GNPs biosynthesis based the cell-free supernatant produced by *S. albogriselus*. GNPs were obtained by treating the HAuCl_4_ aqueous solution with the cell-free supernatant produced by *S. albogriselus*. To avoid photolytic reactions, the incubation was carried out in a complete darkness using an incubator shaker. Following the incubation period, the synthesis of GNPs was initially indicated by a colour change to red or purple (Fig. 1A). Consequently, HAuCl_4_ can be reduced to the appropriate GNPs by the cell-free supernatant produced by *S. albogriselus*. The color change of GNPs solution is explained by surface plasmon resonance (SPR) hypothesis and due to the size variation of the NPs.

Surface plasmon resonance (SPR) is a phenomenon that occurs when light interacts with a metallic surface. Photons of light transfer their energy to packets of electrons known as plasmons on the surface of a metal. SPR is the resonant oscillation of electrons in the layer of metal surfaces that are activated by incident light photons at a specific angle of incidence and move parallel to the metal surface.Larger NPs are indicated by the absorbance peak at a higher wavelength. The GNPs shape and size depend on the synthesis conditions such as incubation time, pH, temperature, metal salt concentration, as well as plant extract concentration^33^. GNPs' optical properties vary from pale pink to deep red based on their shape and size, as well as their degree of aggregation^34^.

A diverse range of microorganisms (including bacteria, fungi, algae, and actinomycetes) have been used as effective eco-friendly agents for metal NPs biosynthesis because of their remarkable advantages such as easy processing and management, lower cost of medium needed for their growth, the ability to reduce and stabilize biogenic compounds^35,36^. While a large number of microbial species are capable of producing metal NPs, the mechanism of NPs biosynthesis has not been established. The metabolic complexity of viable microorganisms complicates the analysis and identification of active species involved in the nucleation and growth of metal NPs. The biosynthesis of GNPs occurs in two steps: first, reducing agents convert gold ions (Au^3+^) into GNPs (Au^0^), and then GNPs are capped with biological molecules^14,37^. Depending on where GNPs are produced, it has been found that the biosynthesis pathway for bacteria is both extracellular and intracellular. However, the most common mechanism for biosynthesis of GNPs is extracellular synthesis. It is assumed that the enzymatic process is one of the most effective ways for biosynthesis of GNPs. GNPs biosynthesis in bacteria, like *Pseudomonas fluorescence*, is associated with the enzyme NADPH-dependent reductase, which converts Au^3+^ to Au^0^ via an enzymatic metal reduction process including electron transfer^14^. Numerous biological components (including sugars, phenols, enzymes, and others) can participate both in the reduction of gold into GNPs and in the stabilizing and capping of NPs. The participation of various biomolecules in the GNPs biosynthesis the functionalities^14^.

### UV–visible spectrum analysis of GNPs

GNPs biosynthesis was investigated by the use of UV–visible spectroscopy throughout the spectral range of 300–800 nm (Fig. 1B). The results indicate that the maximum absorption peak observed is located at 540 nm. Song et al.^38^ declared that the quantitative measurement of the GNPs concentration was recorded at 540 nm. Khadivi Derakhshan et al.^39^ stated that the UV visible absorption spectrum of GNPs synthesized using cells of *Streptomyces griseus* showed a strong and broad peak at 540 nm. The biosynthesis of GNPs using marine *Streptomyces griseus* was confirmed by the transformation of the yellow colour of gold cations in HAuCl_4_ solution into the pink colour of the colloidal GNPs and the maximum absorption peak was observed at 534 nm^40^. However, Biglari et al.^41^ reported that the UV–vis absorption spectrum of GNPs biosynthesised by *Streptomyces djakartensis* isolate B-5 exhibited a single peak with a maximum absorption at 530 nm. On the other hand, Kalabegishvili et al.^42^ reported that the maximum absorption peak of GNPs biosynthesised by *Streptomyces glaucus* 71MD biomass was observed at 530 nm. The maximum absorption peaks of GNPs synthesized using leaf extracts of *Annona muricata* occurring were observed at 530 and 538 nm^43^. The peak of the SPR of GNPs in an aqueous solution change to longer wavelengths as the particle size increases. Mie^44^ states that spherical NPs should only have one SPR band in their absorption spectra, while anisotropic particles may have two or more SPR bands, depending on their form.

### Microscopy analysis of GNPs

**Figure 2:**
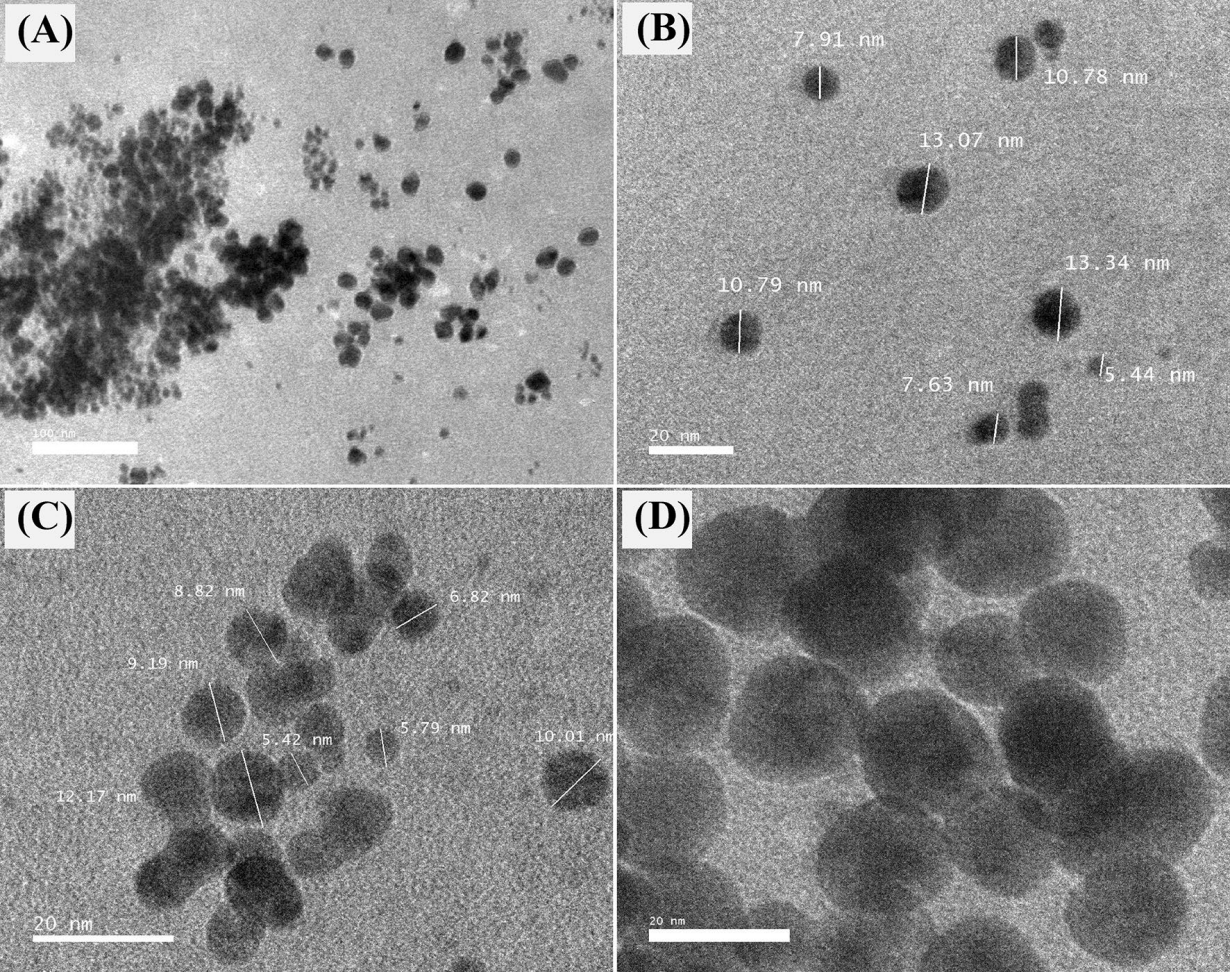
TEM photos of GNPs biosynthesized by *S. albogriseolus* (**A**–**D**). TEM images at a resolution of 20–100 nm and accelerating voltage of 200 kV have been used.

**Figure 3:**
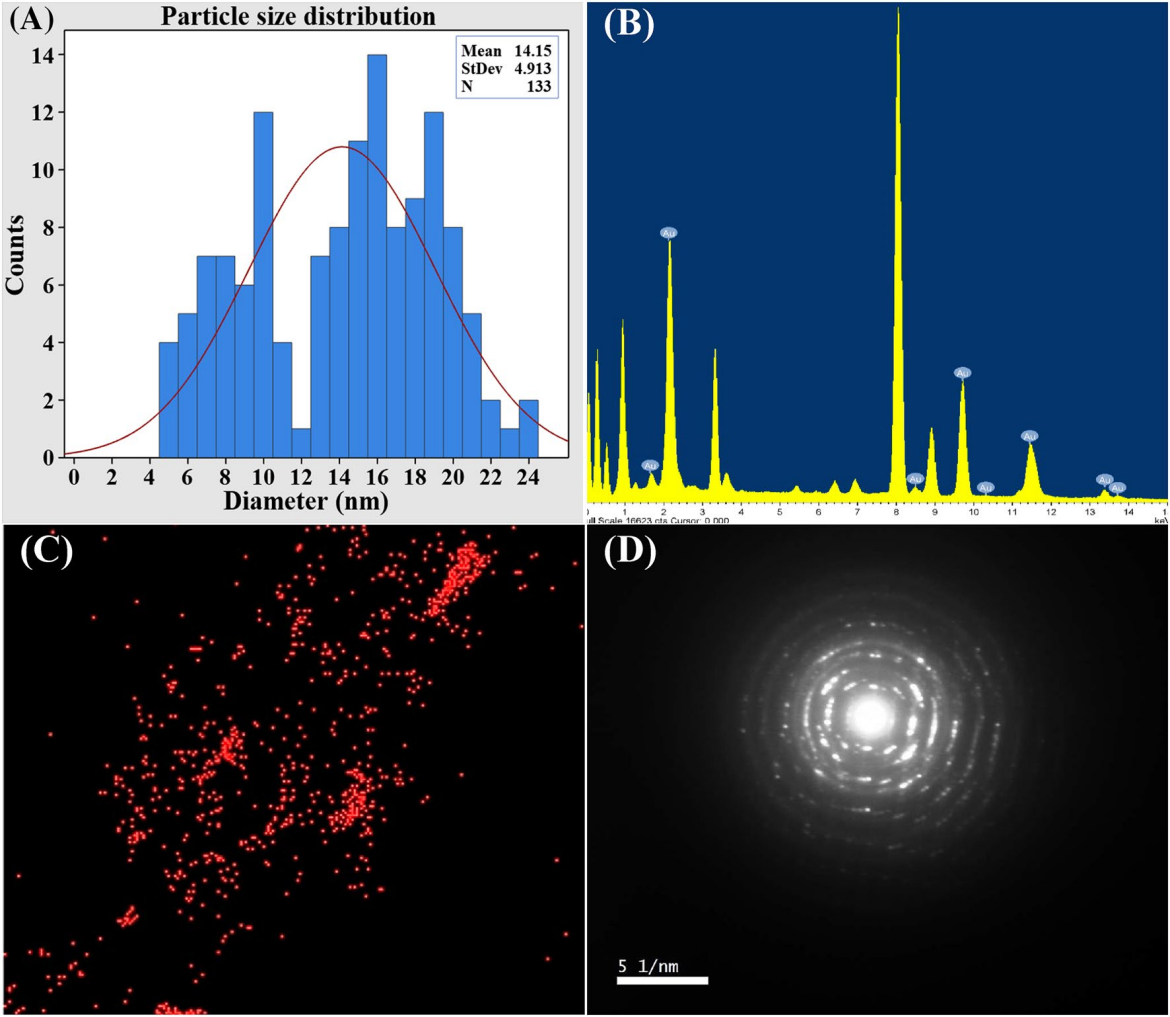
Particle size distribution (**A**), EDX analysis (**B**), mapping analysis (**C**) and selected area electron diffraction (SAED) (**D**) of the biosynthesized GNPs.

TEM examination was used to assess the size and shape of the biosynthesized GNPs obtained from the experimental trial numbered 21, which had the highest yield of the biosynthesized GNPs. Figure 2A–D demonstrate the formation of well-dispersed spherical GNPs with sizes ranging from 5.42 to 13.34 nm. Singh et al. reported that TEM image depicted that the biosynthesized GNPs by *Panax ginseng* leaves mainly have spherical shapes with particle size in the range of 3.41–14.5^45^. The histogram in Fig. 3A displays the distribution of particle sizes, which was determined by analyzing 133 particles with mean size of 14.15 nm. The GNPs synthesized by cell-free supernatant of *Streptomyces cyaneus* strain Alex-SK121 were examined using TEM, which revealed spherical particles with nano sizes ranging from 16.4 to 26.6 nm^46^. Previous studies have documented the synthesis of GNPs using *Pseudomonas denitrificans*, yielding NPs within the size range of 5–25 nm^47^. The triangular and spherical forms of GNPs have been verified by TEM images, indicating a consistent and homogeneous particle composition. Mahdi and Parveen^48^ reported that the average particle size of GNPs varied from 15 to 20 nm.

Figure 3B showed the EDX analysis of the biosynthesized GNPs. A widely used analytical technique for determining the elemental composition of a particular material is the EDX analysis^49^. Figure 3C showed TEM elemental mapping analysis of the biosynthesized GNPs. The mapping analysis result illustrate the distribution and the composition of the biosynthesized GNPs.

The crystallinity of the biosynthesized GNPs was evaluated using TEM- selected area electron diffraction pattern (SAED) analysis (Fig. 3D). SAED patterns often produce an image of single spots (dots) for any crystalline nanomaterial that is a single crystal, ring pattern, or polycrystalline material. This unique SAED property is important for distinguishing between crystalline and amorphous nanomaterials. The crystalline structure of GNPs was confirmed by the observation of distinct, bright, circular well-defined diffraction rings. These rings are related to four specific lattice planes: (111), (200), (220), and (311). The presence of these diffraction patterns indicated the fcc (face-centered cubic) structure of gold^50^.

### Fourier transformed infrared analysis (FTIR)

**Figure 4:**
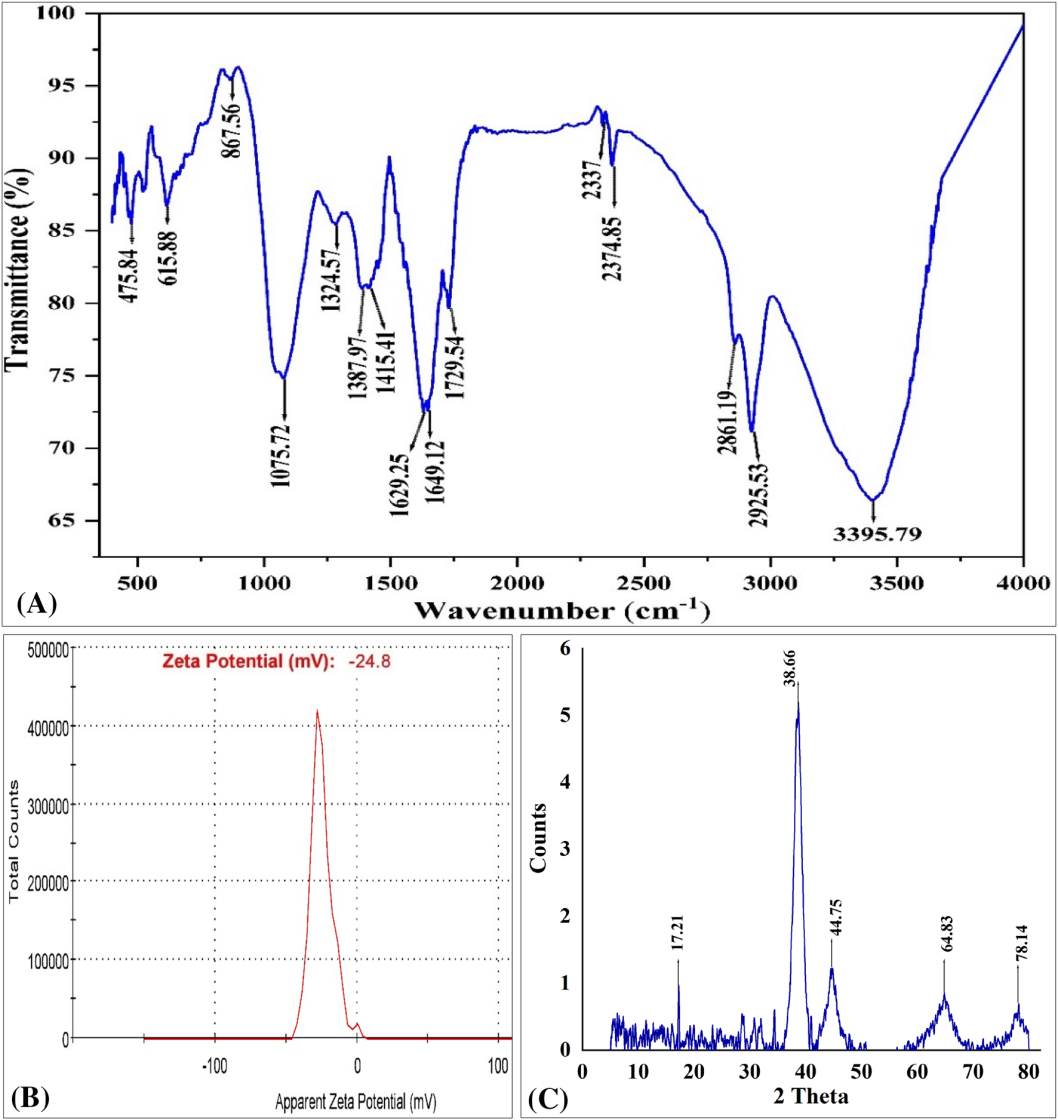
Analyses of the biosynthesized GNPs with FTIR (**A**), zeta potential (**B**), and XRD (**C**).

Fourier-transformed infrared spectroscopy is used to study the chemical composition of the GNPs' surface, to identify the functional groups and the capping agents on the NPs^51^. FTIR spectroscopy was used to identify the biomolecules that are responsible for both capping and effective stability of the metal NPs^52^. The FTIR spectrum of GNPs (Fig. 4A) reveals fifteen absorption peaks at 3395.79, 2925.53, 2861.19, 2374, 2337, 1729, 1649, 1629, 1415, 1387, 1324, 1075, 867.56, 615.88, and 475.84. The FTIR peak at 3395.79 cm^−1^ is attributed to the presence of O–H bonds in the aromatic, alcoholic, and phenolic compounds^53^. The FTIR peak at 2925.53 cm^−1^ in the spectra of DLP-GNPs indicated the presence of the C–H group^54^. The stretching bond of carbonyl group connected to the amide linkage is responsible for the amide band I, which has a distinctive band at 1649 cm^−1^^55^. The peak at 1387 cm^−1^ is corresponding to aromatic amine group (C–N) stretch bond^56^. The peak at 1072 cm^−1^ was indicative of amines^57^. The peak at 867.56 cm^−1^ correlates to C–H stretch bond which is indicative of alkenes' functional group^58^. The bands located at 641 and 475 cm^−1^ are due to the presence of amide group^59^.

### Zeta potential analysis of GNPs

The NPs' net charge is a crucial factor that significantly impacts their dispersion and stability properties (El-Naggar et al.^60^). Therefore, Zeta potential values were used to determine the biosynthesized GNPs stability and their surface charge. The measurement of zeta potential is based upon particle velocity and direction within a well controlled electric field^61^. The results indicate that the biosynthesized GNPs showed zeta potential value of − 24.8 mV (Fig. 4B). The capping molecules that are present on the surface of GNPs are primarily composed of negatively charged groups^61^. Particles in suspension will repel each other and not aggregate if their zeta potential is large, either positive or negative. On the other hand, there is no force preventing particles with a low zeta potential from flocculating and aggregating^23^. The negative zeta potential value implies that GNPs are bounded by negatively charged organic molecules, which decreases the repulsion between the GNPs, prevents their aggregation, and eventually promotes their stability^62^. Based on Muthuvel et al.^61^ results, the capping agent found on the surface of GNPs are primarily composed of negatively charged groups and are also responsible for the NPs stability. Dutta et al.^63^ observed that zeta potential value of the GNPs synthesized by leaf of *S. jambos* was − 28 mV. A significant negative zeta potential value means a high stable conditions for nanoparticle-containing solutions. Omolaja et al.^64^ reported that the zeta potential values range from + 100 mV to − 100 mV. Prashanth and Onkarappa^65^ stated that the GNPs had a zeta potential of − 23.2 mV, which demonstrated the stability and monodispersion of the synthesized GNPs in the colloidal solution.

### X-Ray diffraction pattern (XRD)

The biosynthesized GNPs using the cell-free supernatant of *S. albogriseolus* was analyzed using XRD pattern (Fig. 4C). In the range of 0–80°, the peaks of GNPs were observed at 2*θ* = 17.21°, 38.66°, 44.75°, 64.83°, and 78.14° which coordinate with the results of Skladanowski et al.^66^. Doan et al.^50^ reported that the synthesized GNPs were crystalline in nature and that the maximum diffraction peak occurs at a 2*θ* angle of around 38.2°, indicating that the crystals had a preferential growth orientation in the plane (111). The X-ray diffraction technique is regarded as a fundamental characterisation tool in NPs research for determining critical properties such as crystal structure and crystallite size. The absence of total constructive and destructive X-ray interferences in a finite sized lattice causes the randomly oriented crystals in nanocrystalline materials and broadening of diffraction peaks^67^. The Debye–Scherrer’s formula, *d* = *K*λ/β*cosθ*, is the most widely used method for estimating the crystallite size from the full width at half maximum (FWHM) of a diffraction peak broadening^68^. Where *d* is the nanoparticles crystalline size in nm, λ is the X-ray wavelength (λ = 1.540595 Å), β is the full width at half maximum (FWHM) of the diffraction peak, *θ* is the angle of diffraction and *K* is the Scherrer constant. The crystalline size was estimated from XRD peaks using Debye–Scherrer’s formula, which was 53.359 nm. The values of mean size of NPs, obtained by X-ray diffraction and dynamic light scattering, exhibited a big difference between the two values due to the high agglomeration and random shape of the particles. The most accurate results of the mean particle size were achieved by the TEM. TEM results based on direct measurement of the size of individual NPs. Electron microscopy can distinguish the shape and surface structure of particles, as well as their geometric dimensions, by measuring the width of individual particles in an image^69,70^. Imaging was preferred due to its high-resolution particle imaging and the negligible impact of defects on size measurement^70^.

### Central composite design for GNPs optimization

The properties of metal-NPs produced by a biosynthetic approach are influenced by different variables, including the nature of the biological source used, temperature, pH, reaction media, etc., so optimizing those variables provides control over the NPs' size, shape, and monodispersity^5^. During NPs biosynthesis, stabilizing or capping agents are necessary to minimize their toxicity and improve their biocompatibility and bioavailability in living cells, which improves the biomedical activity of the NPs. Furthermore, the capping agents prevent NPs from aggregating, improve their ability to stay colloidal, and prevent the uncontrolled growth of NPs (particularly those made of metal and metal oxide)^71^. The different forms of capping agents have an effect on the particle's shape, size, catalytic, optical, and magnetic properties.

In the present study, GNPs can vary in size distribution, and quantity during the biosynthesis process due to the effects of four independent variables including the HAuCl_4_ concentration, initial pH level, CFS concentration (%), and incubation time. The optimal values of those four variables were obtained through the use of CCD involving a total of 30 experimental runs. Table 1 displays the actual and coded levels of the four variables, the experimental and predicted values for GNPs, accompanied by their corresponding residuals. According to the CCD results of El-Naggar et al.^25^, the GNPs produced using the cell-free supernatant of *Streptomyces flavolimosus* showed notable changes in GNPs color as a result of the optimal levels of various independent variables during the optimization process. Based on the variance of the four variable levels, the results revealed that the biosynthesized GNPs exhibited a range of values, beginning from 190.93 to 778.74 µg/mL. The highest yield of biosynthesized GNPs was 778.74 µg/mL was obtained in the experimental trial numbered 21 with an initial pH of 7, CFS concentration of 70%, HAuCl_4_ concentration of 800 µg/mL, and the incubation time of 96 h. On the other hand, the minimum yield of the biosynthesized GNPs (190.93 µg/mL) was obtained at trial numbered 8 with HAuCl_4_ concentration of 200 µg/mL, an initial pH level of 8, CFS concentration of 80%, and an incubation time of 72 h.

**Table 1:** Central composite design of four variables with their coded and actual levels, mean experimental and predicted results of extracellular biosynthesis of GNPs by using *S. albogriseolus*.

Std	Run	Type	X_1_	X_2_	X_3_	X_4_	GNPs biosynthesis (µg/mL)					
							Actual	CCD		ANN		
								Predicted	Residuals	Predicted	Residuals	Validation
9	1	Factorial	− 1	− 1	− 1	1	335.28	320.03	15.25	335.17	− 0.11	Training
20	2	Axial	0	2	0	0	281.43	294.19	− 12.77	281.96	0.53	Training
29	3	Center	0	0	0	0	425.48	457.47	− 31.99	445.89	20.41	Training
8	4	Factorial	1	1	1	− 1	373.73	382.53	− 8.79	375.16	1.43	Training
28	5	Center	0	0	0	0	459.42	457.47	1.95	445.89	− 13.53	Training
19	6	Axial	0	− 2	0	0	577.08	575.11	1.97	576.51	− 0.57	Training
24	7	Axial	0	0	0	2	496.73	486.9	9.83	497.53	0.80	Training
17	8	Axial	− 2	0	0	0	190.93	217.47	− 26.54	191.36	0.43	Training
1	9	Factorial	− 1	− 1	− 1	− 1	363.89	324.18	39.71	363.89	0.00	Training
16	10	Factorial	1	1	1	1	485.25	520.63	− 35.38	485.49	0.24	Training
5	11	Factorial	− 1	− 1	1	− 1	391.7	420.16	− 28.46	403.14	11.44	Validation
4	12	Factorial	1	1	− 1	− 1	460.49	454.22	6.27	460.24	− 0.25	Training
3	13	Factorial	− 1	1	− 1	− 1	284.77	284.33	0.4333	291.12	6.35	Training
25	14	Center	0	0	0	0	458.19	457.47	0.722	445.89	− 12.30	Validation
23	15	Axial	0	0	0	− 2	332.32	352.95	− 20.62	344.88	12.56	Validation
13	16	Factorial	− 1	− 1	1	1	396.17	398.11	− 1.94	424.43	28.26	Validation
27	17	Center	0	0	0	0	453.27	457.47	− 4.2	445.89	− 7.38	Training
26	18	Center	0	0	0	0	473.36	457.47	15.89	445.89	− 27.47	Validation
14	19	Factorial	1	− 1	1	1	767.72	761.7	6.03	768.18	0.46	Training
22	20	Axial	0	0	2	0	559.45	557.13	2.32	560.08	0.63	Training
10	21	Factorial	1	− 1	− 1	1	778.74	809.89	− 31.15	777.32	− 1.42	Validation
2	22	Factorial	1	− 1	− 1	− 1	659.08	671.76	− 12.68	656.90	− 2.18	Training
12	23	Factorial	1	1	− 1	1	645.14	610.22	34.92	645.13	− 0.01	Training
21	24	Axial	0	0	− 2	0	537.64	550.75	− 13.11	538.08	0.44	Training
7	25	Factorial	− 1	1	1	− 1	374.39	338.91	35.48	362.89	− 11.50	Training
11	26	Factorial	− 1	1	− 1	1	282.31	298.05	− 15.74	294.27	11.96	Validation
6	27	Factorial	1	− 1	1	− 1	661.54	641.46	20.08	643.15	− 18.39	Validation
30	28	Center	0	0	0	0	475.09	457.47	17.62	445.89	− 29.20	Validation
18	29	Axial	2	0	0	0	766.7	750.95	15.75	767.07	0.37	Training
15	30	Factorial	− 1	1	1	1	353.87	334.73	19.14	342.09	− 11.78	Validation

Variable	Code	− 2	− 1	0	1	2						
HAuCl_4_ concentration (µg/mL)	X_1_	200	400	600	800	1000						
Initial pH level	X_2_	6	7	8	9	10						
CFS concentration (%)	X_3_	60	70	80	90	100						
Incubation time (h)	X_4_	24	48	72	96	120						

### Multiple regression analysis and ANOVA

Analysis of variance (ANOVA) and multiple-regression statistical analysis were used to evaluate the relationship between biosynthesized GNPs and independent variables. Table 2 includes the coefficient estimate values, R^2^ value, predicted R^2^ value, adjusted R^2^ value, *P*-values (probability value), lack of fit, and *F*-value (Fisher value), all of which were assessed to confirm the adequacy of the model. In addition, the linear, interaction, and quadratic effects of the four variables were investigated^18^. The model's adequacy was assessed using the determination coefficient (R^2^), which reflects the degree of response value variability that can be attributed to the independent variables^72^. A model is considered more reliable and capable of predicting responses more accurately when its R^2^ value is closer to one. R^2^ values are always less than or equal to 1. A model with an R^2^ values higher than 0.9 was considered to have a very high correlation^73^. In the present research, the R^2^ value of the model used for GNPs biosynthesis by CFS of *S. albogriselus* is 0.9822 (Table 2), indicating that 98.22% of the variability in GNPs biosynthesis could be explained by the model, and only 1.78% of the total variance could not be explained by the model. On the other hand, the adjusted-R^2^ explains the variance in the response as affected by the independent variables, the regression model used for GNPs biosynthesis by CFS of *S. albogriselus* has an adjusted R^2^ of 0.9655. The high value of adjusted R^2^ proves the high model significance. The predicted-R^2^ was calculated in order to estimate how well the model predicts the response for new experiments^74^. The predicted R^2^ (pred. R^2^) value of 0.9075 demonstrated satisfactory agreement with the adj. R^2^ value, indicating a strong agreement between the observed and predicted response values of GNPs biosynthesis, thus the model is useful for predicting GNPs biosynthesis in subsequent experiments. The great values of predicted R^2^ and adjusted R^2^ indicated a strong correlation between theoretical and experimental values and the high significance of the model^75^.

**Table 2:** Analysis of variance for extracellular biosynthesis of GNPs by using *S. albogriseolus* as affected by HAuCl_4_ concentration (µg/mL), initial pH level, CFS conc. (%), and incubation time (h).

Source of variance		Coefficient estimate	Sum of squares	Degrees of freedom	Mean square	*F*-value	*P*-value
Model	Intercept	457.47	665,500	14	47,533.24	59.02	< 0.0001*
Linear effect	X_1_	133.37	426,900	1	426,900	530.02	< 0.0001*
	X_2_	− 70.23	118,400	1	118,400	146.97	< 0.0001*
	X_3_	1.6	61.17	1	61.17	0.076	0.7866
	X_4_	33.49	26,913.47	1	26,913.47	33.42	< 0.0001*
Interaction effect	X_1_ X_2_	− 44.42	31,573.74	1	31,573.74	39.2	< 0.0001*
	X_1_ X_3_	− 31.57	15,944.75	1	15,944.75	19.8	0.0005*
	X_1_ X_4_	35.57	20,244.51	1	20,244.51	25.14	0.0002*
	X_2_ X_3_	− 10.35	1713.98	1	1713.98	2.13	0.1652
	X_2_ X_4_	4.47	319.29	1	319.29	0.3964	0.5384
	X_3_ X_4_	− 4.47	320.34	1	320.34	0.3977	0.5378
Quadratic effect	X_1_^2^	6.69	1226.10	1	1226.10	1.52	0.2363
	X_2_^2^	− 5.7	892.29	1	892.29	1.11	0.3092
	X_3_^2^	24.12	15,955.32	1	15,955.32	19.81	0.0005*
	X_4_^2^	− 9.39	2416.43	1	2416.43	3	0.1038
Error effect	Lack of Fit		10,473.28	10	1047.33	3.26	0.1023
	Pure Error		1608.14	5	321.63		
R^2^	0.9822						
Adj R^2^	0.9655						
Pred R^2^	0.9075						
Adeq Precision	29.52						

*Significant values, *F* Fishers’s function, *P* level of significance.

In Table 2, *F*-values and probability values (*P*-values) were used to assess the significance of various coefficients and to understand their interactions. The coefficient's significance increased as its *P*-value decreased. Additionally, process variables with *P*-values equal to or less than 0.05 were regarded as having a significant impact on the response^76^. The model's *F*-value (59.02) and *P*-value (< 0.0001) imply it is highly significant. A *P*-value of < 0.05 suggests the significance of linear coefficients in the biosynthesis of GNPs. Based on the *P*-values in Table 2, the linear effects of HAuCl_4_ conc. (X_1_), the initial pH level (X_2_), and incubation time (X_4_) are significant for GNPs biosynthesis by *S. albogriselus.* X_1_, X_2_, and X_4_ had *F*-values of 530.02, 146.97, and 33.42, and their *P*-values were < 0.0001. This means that the three variables have a significant effect on GNPs biosynthesis. Even minor differences in their levels can cause variations in the GNPs biosynthesis process. According to the *P*-value (*P*-value < 0.05), the interaction effects between X_1_ X_2_, X_1_ X_3_, X_1_ X_4_ have a significant effect on GNPs biosynthesis. Moreover, the sign (positive or negative) of the coefficients has an impact on the response (GNPs biosynthesis). The effects of two-factor interactions can be either synergistic (positive) or antagonistic (negative)^77^. A positive sign is associated with an increase in the response at elevated levels of the variables. Conversely, a negative sign reveals that the response is higher at a lower value of the variables^78^. The linear effects of X_1_ and X_4_, have a positive sign with a *P*-value < 0.0001, which indicates that GNPs production increases when these variables are at their highest levels. On the other hand, X_2_ has a negative sign, which indicates its negative effect on GNPs biosynthesis at a high level. If the coefficient for interactions between two variables is positive, this suggests a synergistic effect arising from the interactions between two the variables on the GNPs biosynthesis. The interaction between variables X_1_ and X_4_ shows a positive coefficient with a *P*-value of 0.0002, suggesting that increasing their values leads to improved GNPs biosynthesis. However, the presence of a negative coefficient shows the existence of an antagonistic impact caused by the interactions between two variables X_1_X_2_ and X_1_X_3_ leads to decreased GNPs biosynthesis. However, the presence of a negative coefficient indicates that the interactions between two variables X_1_X_2_ and X_1_X_3_, have an antagonistic impact, resulting in decreased GNPs biosynthesis. The adequate precision (adq. Precision) of the model was determined to be 29.52, suggesting that the model has appropriate precision for GNPs biosynthesis optimization at various parameter levels. The adq. Precision value indicates the signal-to-noise ratio. A signal-to-noise ratio greater than 4 is desired and indicative of the model's precision^79^.

A second-order polynomial equation was used to investigate the correlation between independent and dependent variables. The highest GNPs biosynthesis was predicted using the second-order polynomial equation based on the used levels of X_1_ (HAuCl_4_ concentration), X_2_ (initial pH level), X_3_ (CFS concentration) and X_4_ (the incubation time). GNPs biosynthesis' prediction (Y) regarding the independent variables (X_1_, X_2_, X_3_ and X_4_) shown in the following second-order polynomial equation:

$$\begin{gathered} {\text{Y}} = {457}.{47} + {133}.{\text{37 X}}_{{1}} - {7}0.{\text{23 X}}_{{2}} + {1}.{\text{6 X}}_{{3}} + {33}.{\text{49 X}}_{{4}} \hfill \\ \quad \quad \; - {44}.{\text{42 X}}_{{1}} {\text{X}}_{{2}} - {31}.{\text{57 X}}_{{1}} {\text{X}}_{{3}} + {35}.{\text{57 X}}_{{1}} {\text{X}}_{{4}} - {1}0.{\text{35 X}}_{{2}} {\text{X}}_{{3}} + {4}.{\text{47 X}}_{{2}} {\text{X}}_{{4}} \hfill \\ \quad \quad \; - {4}.{\text{47 X}}_{{3}} {\text{X}}_{{4}} + { 6}.{\text{69 X}}_{{1}}^{{2}} - {5}.{\text{7 X}}_{{2}}^{{2}} + {24}.{\text{12 X}}_{{3}}^{{2}} - {9}.{\text{39 X}}_{{4}}^{{2}} \hfill \\ \end{gathered}$$

Y represents the GNPs biosynthesis’ prediction, X_1_ represents the HAuCl_4_ concentration, X_2_ represents the initial pH, X_3_ represents CFS concentration, and X_4_ represents the incubation time.

The fit summary results presented in Table 3 were employed to assess the most suitable polynomial models amongst linear, 2FI, and quadratic for the biosynthesis of GNPs using the cell-free supernatant of *S. albogriselus*. The quadratic model is an appropriate model for GNPs biosynthesis since the lack of fit is non-significant (*P*-value is 0.1023 and the *F*-value is 3.26) (Table 3). Furthermore, the quadratic model has a higher R^2^ value (0.9822), adjusted R^2^ value (0.9655), and predicted R^2^ value (0.9075) than other models. Furthermore, quadratic model summary statistics for GNPs biosynthesis showed a minimum standard deviation of 28.38 and a minimum PRESS value of 62,641.8. If the PRESS statistic is low, it indicates that the model is valid and can fit the data adequately.

**Table 3:** Fit summary for central composite design results for extracellular biosynthesis of GNPs by using *S. albogriseolus* as affected by HAuCl_4_ concentration (µg/mL), initial pH level, CFS conc. (%) and incubation time (h).

Fit Summary					
Source	Sequential *P*-value	Lack of fit *P*-value	Adjusted R^2^	Predicted R^2^	
Linear	< 0.0001*	0.003*	0.8197	0.759	
2FI	0.0009*	0.0183*	0.9207	0.8674	
Quadratic	0.0019*	0.1023	0.9655	0.9075	

Sequential Model Sum of Squares					
Source	Sum of squares	*Df*	Mean square	*F-*value	*P*-value
Linear vs mean	572,200	4	143,100	33.96	< 0.0001*
2FI vs linear	70,116.61	6	11,686.1	6.31	0.0009*
Quadratic vs 2FI	23,114.91	4	5778.73	7.17	0.0019*

Lack of Fit Tests					
Source	Sum of squares	*Df*	Mean square	*F-*value	*P*-value
Linear	103,700	20	5185.24	16.12	0.003*
2FI	33,588.19	14	2399.16	7.46	0.0183*
Quadratic	10,473.28	10	1047.33	3.26	0.1023

Model Summary Statistics					
Source	Std. dev.	R^2^	Adjusted R^2^	Predicted R^2^	PRESS
Linear	64.9	0.8446	0.8197	0.759	163300
2FI	43.04	0.9481	0.9207	0.8674	89833.66
Quadratic	28.38	0.9822	0.9655	0.9075	62641.8

*Significant values, *df* degree of freedom, *2FI* two factors interaction.

### The model’s adequacy assessment

**Figure 5:**
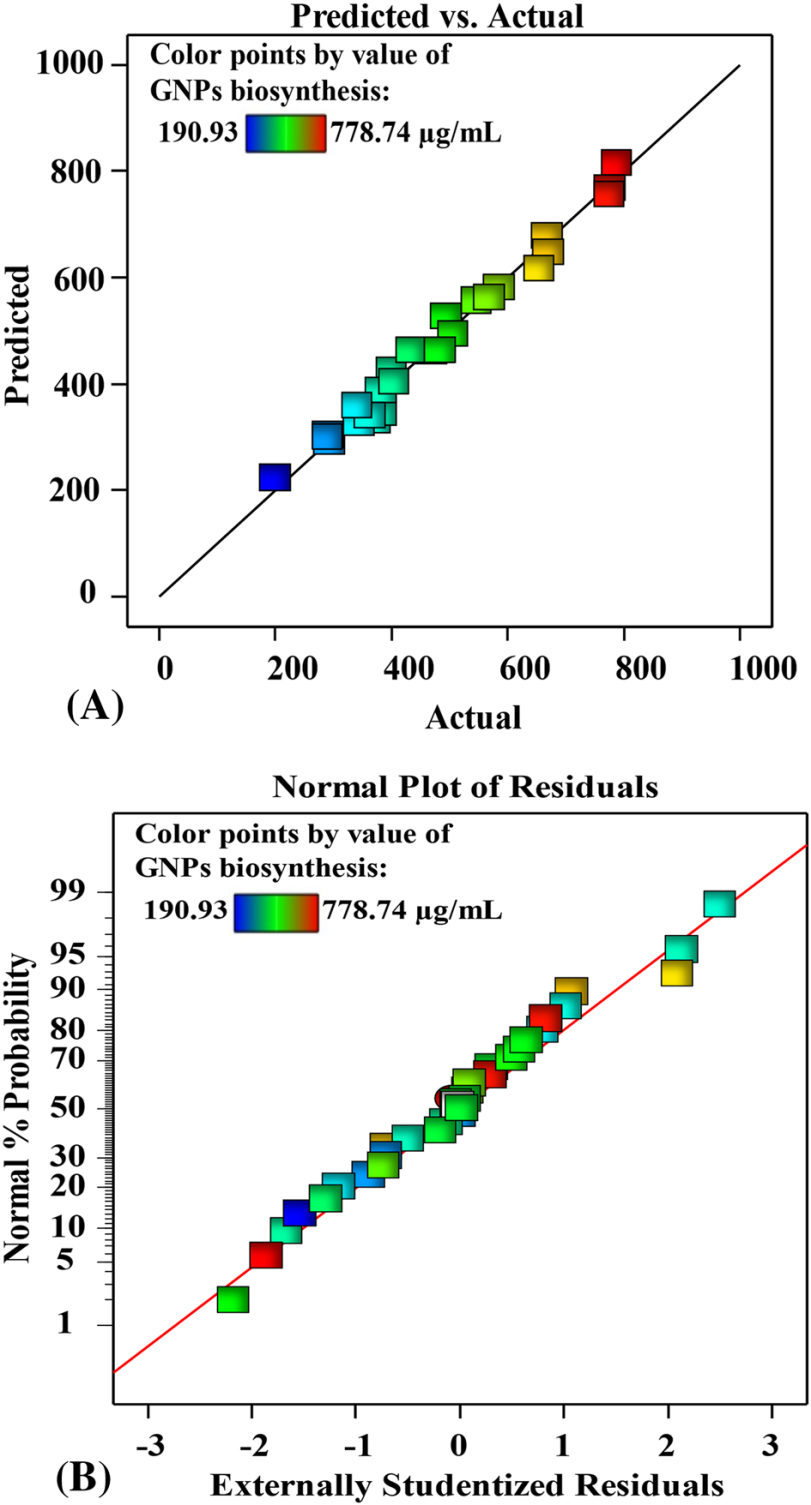
(**A**) Plot of predicted versus actual (**B**), NPP of internally studentized residuals of the biosynthesized GNPs using the cell-free supernatant of *S. albogriseolus*.

Figure 5A compares the actual values with the predicted values by the model for the biosynthesis of GNPs using the cell-free supernatant of *S. albogriselus*. This plot demonstrates that each data point is positioned closely to the prediction line, indicating a strong match between the actual values and the predicted values and confirming the validity of the model^2^. The normal probability plot (NPP) is an essential diagnostic tool that shows if the residuals have a normal distribution, and confirming the fitness of the model^1^. The externally studentized residuals are displayed against the normal % probability. The residuals are observed to be present along the diagonal straight line of GNPs biosynthesis (Fig. 5B). This illustrates that the predicted results fit well with the experimental results, indicating that the model is appropriate.

### Three-dimensional plot (3D)

**Figure 6:**
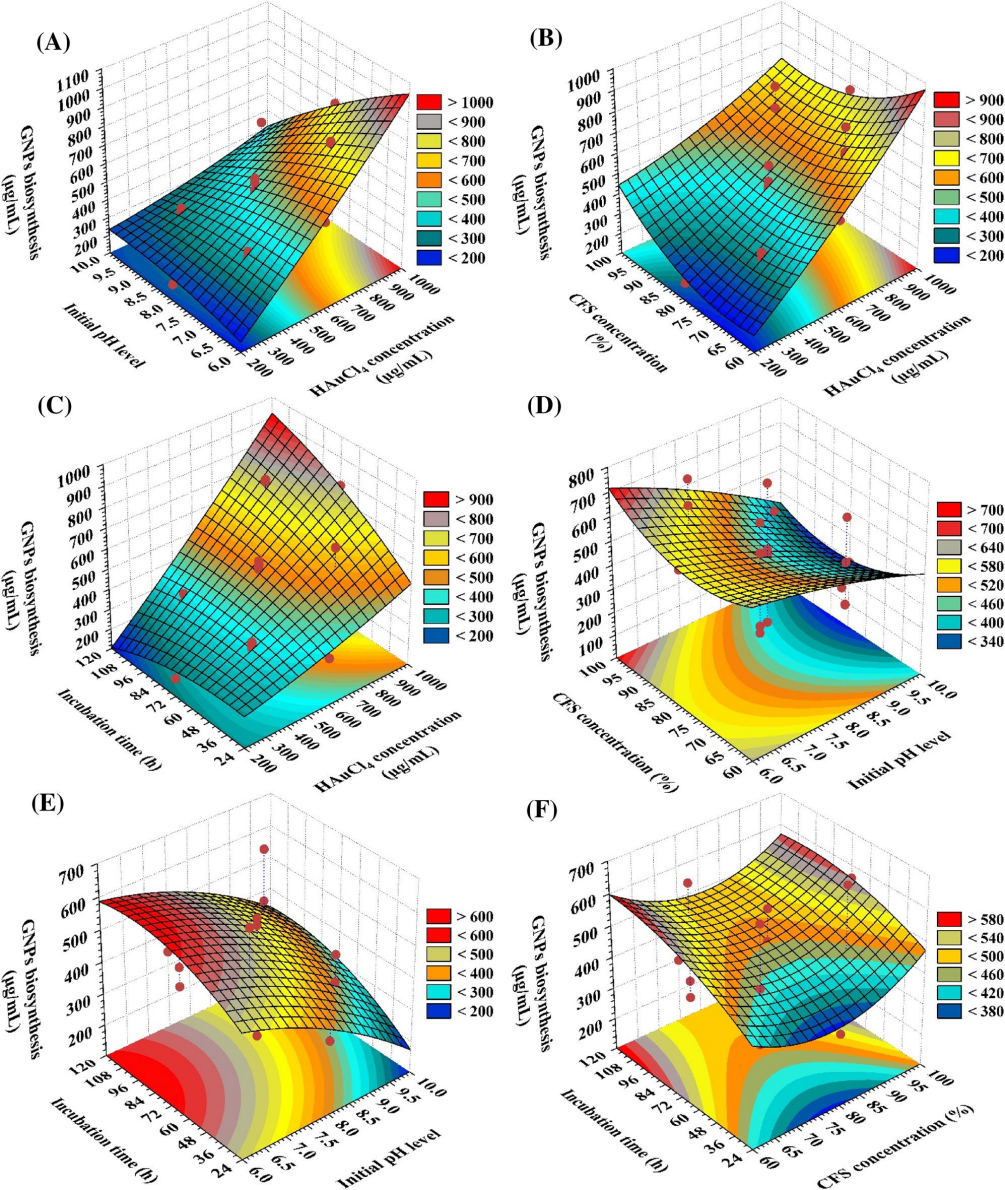
3D plots of the biosynthesized GNPs using *S. albogriseolus* cell-free supernatant, showing the mutual interactions effects of the tested variables.

In order to find the optimal conditions for the biosynthesis of GNPs using the cell-free supernatant of *S. albogriseolus*, the relationship between the biosynthesized GNPs and the mutual interactions between the variables that were evaluated is depicted in Fig. 6A–F. 3D graphs were generated for the pair-wise combinations of the four variables including HAuCl_4_ concentration (X_1_), the initial pH (X_2_) the CFS concentration (X_3_) and incubation time (X_4_). The data points were obtained by plotting the biosynthesis of GNPs on the Z-axis against the X and Y axes for two independent variables. The values of the remaining two variables were kept constant at their respective central points.

Figure 6A illustrates the mutual interactions between HAuCl_4_ concentration (X_1_) and the initial pH (X_2_) while maintaining the CFS concentration (X_3_) and incubation time (X_4_) at their center values for the biological synthesis of GNPs. It demonstrates that the largest amount of the biosynthesized GNPs was produced when the pH level was at its optimal level (7). The concentration of HAuCl_4_ strongly influences the synthesis of GNPs. It can be observed that the biosynthesized GNPs steadily increased as the concentration of HAuCl_4_ increased near to 800 µg/mL. When the concentration of HAuCl_4_ is reduced, the biosynthesized GNPs decrease. The linear effects of HAuCl_4_ conc. (X_1_) and the initial pH level (X_2_) are significant for GNPs biosynthesis by *S. albogriselus*. However, the interactions between the two variables, X_1_X_2_, leads to decreased GNPs biosynthesis as the coefficient sign is negative (Table 2).

### The effect of pH on GNPs biosynthesis

In the present investigation, the optimal pH for maximum biosynthesis of GNPs using the cell-free supernatant of *S. albogriselus* was 7. Zonooz et al.^80^ reported that the optimal pH for the highest biosynthesis of GNPs by the supernatant of *Streptomyces* sp. ERI-3 was 6. When the pH of the aqueous solution was increased from 3 to 9, it changed colours to dark pink, light pink, orange, red, dark purple, greenish-blue, and green^80^. The highest biosynthesis of GNPs by microbial cells normally occurs in the pH range of 2–6, and the variations in the pH of the medium influenced the particle count, dimension, and morphology^81^. On the other hand, Hammami et al.^33^ reported that the GNPs' shapes vary according to the medium's pH: pH 2 results in larger, rod-shaped particles; pH 3–4 produces smaller, rod-shaped particles; pH 8 produces spherical, oval, polyhedral particles; pH 9 produces spherical particles; pH 10 produces rod-shaped particles; and pH 11 produces nano-wires. The reduction process is accelerated by the progressive increase in pH, leading to the formation of GNPs^82^. The biosynthesis of GNPs by *Aspergillus terreus* is regulated in response to alterations in pH. When the pH is adjusted to 8, the shape of GNPs changes from a spherical to a rod-like structure, with a size distribution ranging from 20 to 29 nm. The GNPs by *Aspergillus terreus* exhibit a spherical morphology under the condition of pH 10, with an average size distribution ranging from 10 to 19 nm^83^.

### Effect of HAuCl_4_ concentration on GNPs biosynthesis

GNPs biosynthesis increased as the concentration of gold chloride increased. It could be attributed to the high HAuCl_4_ concentration, which made more Au^+3^ ions available for reduction to GNPs^84^. Zonooz et al.^80^ stated that GNPs biosynthesis using the cell-free supernatant of *Streptomyces* sp. ERI-3 was obtained using a 3 mM HAuCl_4_ solution. However, GNPs biosynthesis was reduced at concentrations of 3.5 and 4 mM HAuCl_4_ solution. This reduction is most likely due to the toxicity of metal ions on the components used in GNPs synthesis. According to Ranjitha and Rai^3^ study, the average size distribution of the biosynthesised GNPs using DSL was found to be 80.9 nm after adding 3 mL of the supernatant of *Streptomyces griseoruber* was added to the 7 mL of 1 mM Hydrogen tetrachloroaurate (III) (HAuCl_4_. 3H_2_O). Składanowski et al.^66^ employed a 1:1 (v/v) ratio of 3 mM of HAuCl_4_ with the cell-free supernatant of *Sreptomyces* sp. NH21. The majority of the GNPs had a spherical form and measured 10 nm (± 14). Using RSM on GNPs biosynthesis using the *Streptomyces* sp. M137-2 supernatant, the optimum level of HAuCl_4_ was found to be 1 mM. Hamed and Abdelftah^40^ used a 50- mL aqueous solution of 2 mM HAuCl_4_ were treated with 50 mL of the *marine Streptomyces griseus* (M8) supernatant.

Figure 6B illustrates the interactions between the HAuCl_4_ concentration (X_1_), the CFS concentration (X_3_), and the biosynthesized GNPs. while keeping the incubation time (X_4_) and initial pH level (X_2_) at their midpoints. The maximum yield of biosynthesized GNPs was found at a CFS concentration of approximately 70%, and further increases in CFS concentration reduce biosynthesized GNPs yield. Also, it can be observed that the biosynthesized GNPs steadily increased as the concentration of HAuCl_4_ increased near to 800 µg/mL. When the concentration of HAuCl_4_ is reduced, the biosynthesized GNPs decrease. The linear effect of HAuCl_4_ conc. (X_1_) is significant for GNPs biosynthesis by *S. albogriselus* and the linear effect of CFS concentration is non-significant. However, the interaction between the two variables, X_1_X_3_, is significant and leads to decreased GNPs biosynthesis as the coefficient sign is negative (Table 2).

### Effect of reductant agent concentration on GNPs biosynthesis

GNPs have a wide variety of shapes, with spherical NPs being the most common type. The following are some possible shapes of GNPs, depending on the production method: wells, stars, nanorods, cells, hexagons, octahedrons, and triangles^85^. Moreover, a relationship was discovered between the concentration of the extract as reductant agent and the predominant type of GNPs: more triangular and prismatic NPs are formed than hexagonal and spherical ones at lower concentrations of the extract used^86^. According to Abirami et al.^87^, different concentrations, namely, 0.5:4.5, 1:4, 1.5:3.5, 2:3, and 2.5:2.5, of cell-free supernatant of *Streptomyces misionensis* PYA9 and HAuCl_4_ (10 M) in a shaker for 24 h in the dark at 120 rpm, revealed that the combination 1:4 displayed an intense brick red color and the GNPs have a size range of 38–43 nm with a spherical and triangular pyramid in shape.

Figure 6C illustrates the correlation between the biosynthesized GNPs and the mutual interactions between the HAuCl_4_ concentration (X_1_) and the incubation time (X_4_), while the initial pH level (X_2_) and CFS concentration (X_3_) were kept at their zero levels. It is clear that as the incubation duration and HAuCl_4_ concentration were increased, the biosynthesized GNPs steadily increased. It can be observed that the biosynthesized GNPs steadily increased as the concentration of HAuCl_4_ increased near to 800 µg/mL. Also, the maximum yield of biosynthesized GNPs was found at incubation time of approximately 96 h, and further increases in the incubation time reduce biosynthesized GNPs yield. The linear effects of both HAuCl_4_ conc. (X_1_) and the incubation time (X_4_) and the interaction between them are significant for GNPs biosynthesis by *S. albogriselus* and leads to increased GNPs biosynthesis as the coefficient signs for the two variables and the interaction between them are positive (Table 2).

### Effect of incubation time on GNPs biosynthesis

In the present investigation, the optimal incubation time for maximum biosynthesis of GNPs using the cell-free supernatant of *S. albogriselus* was 96–120. Zonooz et al.^80^ found that the greater yield of GNPs biosynthesis using the cell-free supernatant of *Streptomyces* sp. ERI-3 was achieved after 96 h of incubation. On the other hand, the optimum incubation time for maximum yield of GNPs biosynthesis using the cell-free supernatant of *Streptomyces* sp. M137-2 was achieved after 72 h. Camas et al.^88^ reported that the greater GNPs biosynthesis by *Citricoccus* sp. K1D109 was achieved after 24 h of incubation, and that the biosynthesis rate decreased as the incubation time increased. More hexagonal and triangular GNPs are produced when the incubation is shortened^89^.

Figure 6D illustrates the correlation between the initial pH level (X_2_) and CFS concentration (X_3_), while HAuCl_4_ (X_1_) and incubation time (X_4_) were kept at their default settings. The value of the biosynthesized GNPs increased up to the optimal pH of around (7), and subsequently decreased after the pH level exceeded that. The value of the biosynthesized GNPs increased as the CFS concentration rose to 100%. The linear effect of pH level (X_2_) is significant for GNPs biosynthesis by *S. albogriselus*. While, the linear effect of CFS concentration (X_3_) is non-significant. Furthermore, the interactions between the two variables, X_1_X_4_ are also is non-significant. Figure 6E illustrates correlation the between the initial pH (X_2_) and incubation time (X_4_) while maintaining the HAuCl_4_ concentration (X_1_) and CFS concentration (X_3_) at their zero levels. It is clear that as the initial pH (X_2_) and incubation time (X_4_) were increased, the biosynthesized GNPs steadily increased. Also, the maximum yield of biosynthesized GNPs was found at initial pH of approximately 7 and incubation time near to 96 h, and further increases in both initial pH (X_2_) or incubation time (X_4_) reduce biosynthesized GNPs yield.

Figure 6F shows the interactions between CFS concentration (X_3_) and incubation time (X_4_) on the biosynthesized GNPs when HAuCl_4_ concentration (X_1_) and initial pH level (X_2_) were kept at their zero levels. Lower CFS concentration (X_3_) and higher incubation time (X_4_) enable the maximum levels of GNPs formation. The interaction effects between CFS concentration (X_3_) and incubation time (X_4_) are non-significant for GNPs biosynthesis by *S. albogriselus*.

### ANN modeling prediction for GNPs biosynthesis

**Figure 7:**
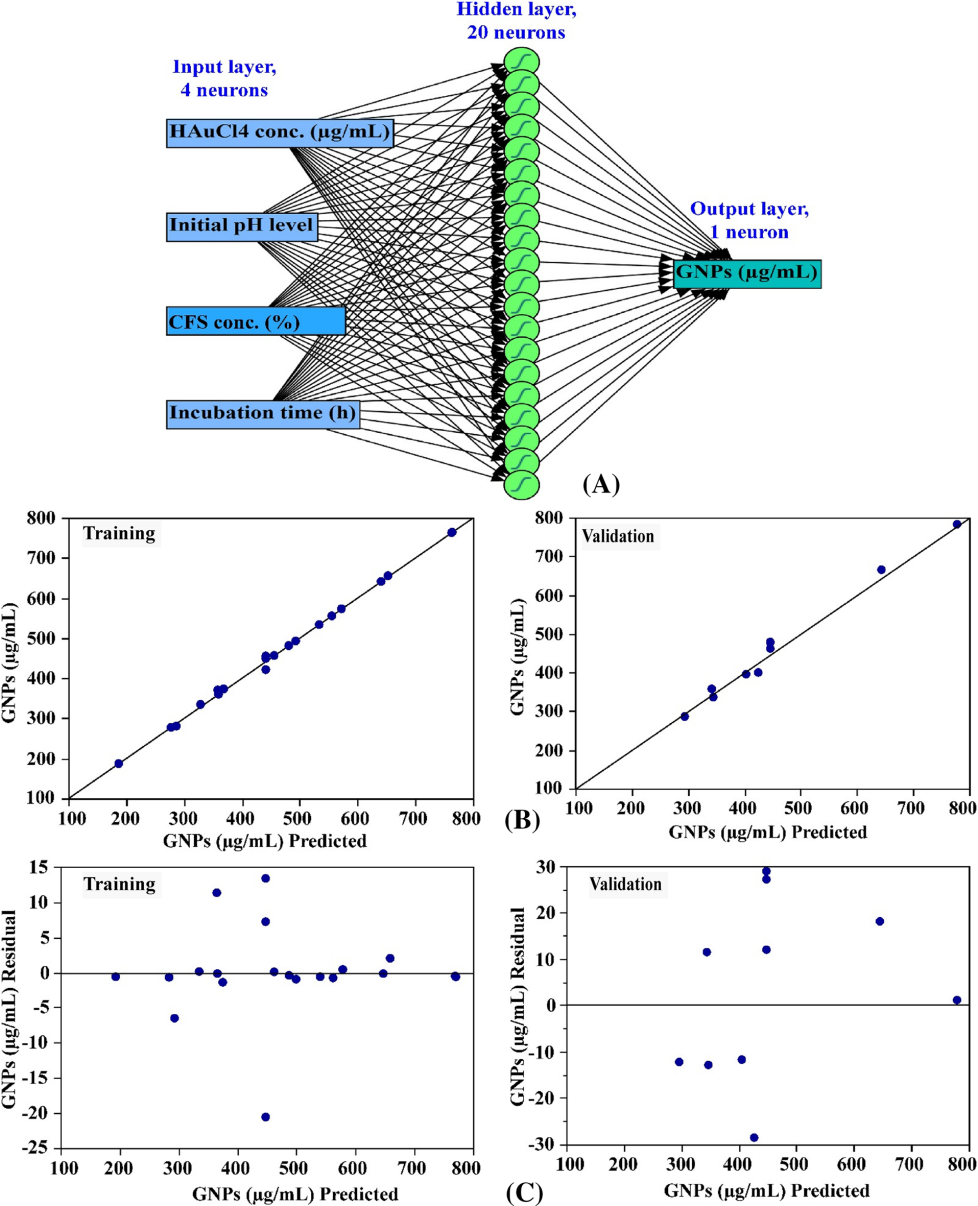
The final artificial neural network of the biosynthesized GNPs (**A**), the ANN predicted versus actual (**B**), and the residuals versus ANN predicted (**C**) values of the biosynthesized GNPs using *S. albogriseolus* cell-free supernatant.

Golnaraghi-Ghomi et al.^90^ applied the artificial neural network (ANN) modeling to find the best conditions for the optimal NP size. They reported that ANN simulated for the optimal NP size and applying ANN method is a useful and cost-effective approach for predicting the results of analysis and modeling of the chemical reactions. To analyze, validate, and predict the GNPs biosynthesis using the cell-free supernatant of *S. albogriseolus*, an artificial intelligence approach was utilized. Artificial Neural Networks (ANN) represent an advanced method within the field of artificial intelligence, facilitating the development of highly effective and dependable computational models, interpreting data, and analyzing it in a manner similar to that of the human mind^25^. Two main factors influence the construction or topology of artificial neural networks: the number of neurons or nodes in each hidden layer and the number of layers. In ANN modelling, the network design includes both learning and training processes, as well as validation and verification of the final ANN model^23^. A simple neural network topology consists of interconnected artificial neurons grouped in three distinct layers: input, hidden, and output^23^. In this study, the input layer accepts the initial data of the four independent variables, namely CFS concentration (%), incubation period (h), HAuCl_4_ concentration (μg/mL), and the initial pH level to be processed by the following layers in the system to predict the optimal conditions for GNPs biosynthesis using the cell-free supernatant of *S. albogriseolus*. The data is sent on to the next layer after being processed, analysed, or categorized by the input nodes. In between the input and output layers is the hidden layer, which consists of 20 neurons. The input layer provides the data to the hidden layer, which transforms it before sending it to the output layer. The output layer presents the final results of the data processing performed by the artificial neural network (GNPs biosynthesis using the cell-free supernatant of *S. albogriseolus*) (Fig. 7A). To achieve optimal performance, the parameters of the ANN were adjusted in the following manner: model NTanH (20), number of tours (5000), a validation method (holdback, 0.2), and a learning rate of 0.1 was used. The sum of squared errors (SSE), the root mean square error (RMSE), and mean absolute deviation (MAD), R^2^ value, for both training and validation processes were determined (Table 4).

**Table 4:** ANN analysis and modeling comparison of predictive capability between CCD and ANN for GNPs biosynthesis using *S. albogriseolus* cell-free supernatant as affected by HAuCl_4_ concentration (µg/mL), initial pH level, CFS conc. (%) and incubation time (h).

Measure	ANN		Overall model performance		
	Training	Validation	Statistics	Measures of fit for CCD	Measures of fit for ANN
R^2^	0.9982	0.9835	R^2^	0.9822	0.9936
RMSE	6.46	18.62	RASE	20.07	11.98
MAD	3.38	16.48	AAE	16.23	7.75
SSE	835.90	3467.82	Freq	30	30
Sum freq	20	10			

*RMSE* the root mean squared error, *MAD* mean absolute deviation, *SSE* the sum of squares error, *RASE* root average squared error, *AA*; average absolute error for each model.

### ANN model evaluation

The ANN was utilized to obtain the predicted results of GNPs in accordance with each individual experimental result, which are displayed in Table 1. A comparison of the ANN model's predictions and the experimental results of the GNPs is shown in Fig. 7B. In both the training and validation processes, the data points are tightly clustered around the line, which represents the best prediction, demonstrating the reliability of the model. Moreover, the distribution of the residual data points is symmetrical, with an equal number of points situated in either direction from the regression line. This suggests that the residuals are dispersed normally and uniformly, as seen in Fig. 7C. This demonstrates that the ANN model is appropriate.

### The prediction potentiality of ANN in comparison with CCD

The purpose of using prediction models (CCD or ANN) was to identify the best values for the variables in order to maximise GNPs biosynthesis yield. The GNPs biosynthesis values predicted by ANN (Table 4) had a higher correlation with the experimental results, and had lower residuals in comparison to the CCD model's residuals. The performance of both CCD and ANN predictions was evaluated by using the model comparison dialogue in JMP Pro14. Various error functions and the coefficient of determination (R^2^) were employed to assess and evaluate the prediction performance of the CCD and ANN models. In Table 4, it is observed that R^2^, root average squared error (RASE), and average absolute error (AAE) are the comparison functions most employed for each regression model. When comparing the prediction capabilities of ANN and CCD, it is shown that ANN has a higher efficiency for accuracy than CCD. That was proved by the higher value of R^2^ (0.9936), as well as the lower values of RASE (11.98) and AAE (7.75), as shown in Table 4. Therefore, it can be concluded that ANN exhibits superior predictive abilities capacity for the optimal levels of GNPs biosynthesis. The observed output can be attributed to the efficacy of ANN to provide good performance can be attributed to the repeated training of the neurons for different physicochemical factors^25^.

### Desirability function (DF)

**Figure 8:**
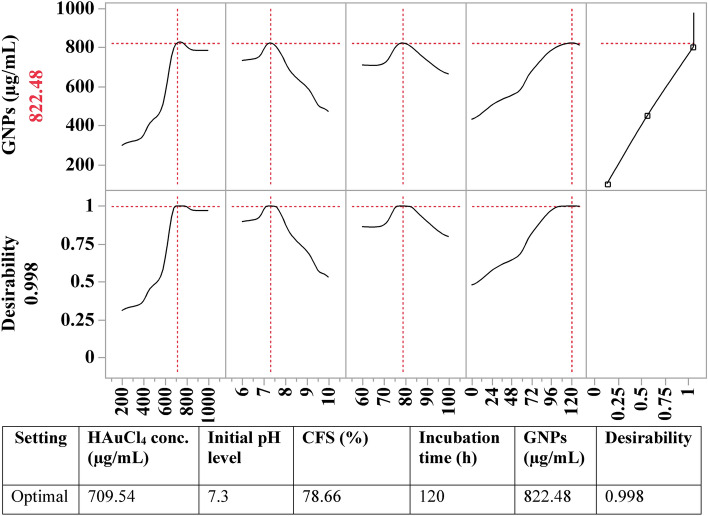
The optimization plot displays the desirability function and the optimum predicted values of the biosynthesized GNPs using *S. albogriseolus* cell-free supernatant.

To determine the optimal predicted conditions for GNPs biosynthesis that would yield the highest value, the desirability function, shown in Fig. 8, was employed. The desirability function of the software Design Expert can be configured for any value between 0 (undesirable) and 1 (desirable)^22^. The desirability function's value is typically calculated theoretically before the optimization procedure is experimentally verified. According to the desirability function, the maximum predicted GNPs biosynthesis using the cell-free supernatant of *S. albogriseolus* was determined to be 822.48 μg/mL under the optimal predicted conditions of HAuCl_4_ concentration (709.54 μg/mL), 7.3 (initial pH level), and a CFS 78.66% at 120 h of incubation. Under these conditions, the maximum experimental value of GNPs biosynthesis using the cell-free supernatant of *S. albogriseolus* was 798 μg/mL. The verification demonstrated that ANN has a high level of accuracy and prediction potential, as the experimental and theoretically predicted values were highly comparable.

### Antitumor activity of GNPs

**Figure 9:**
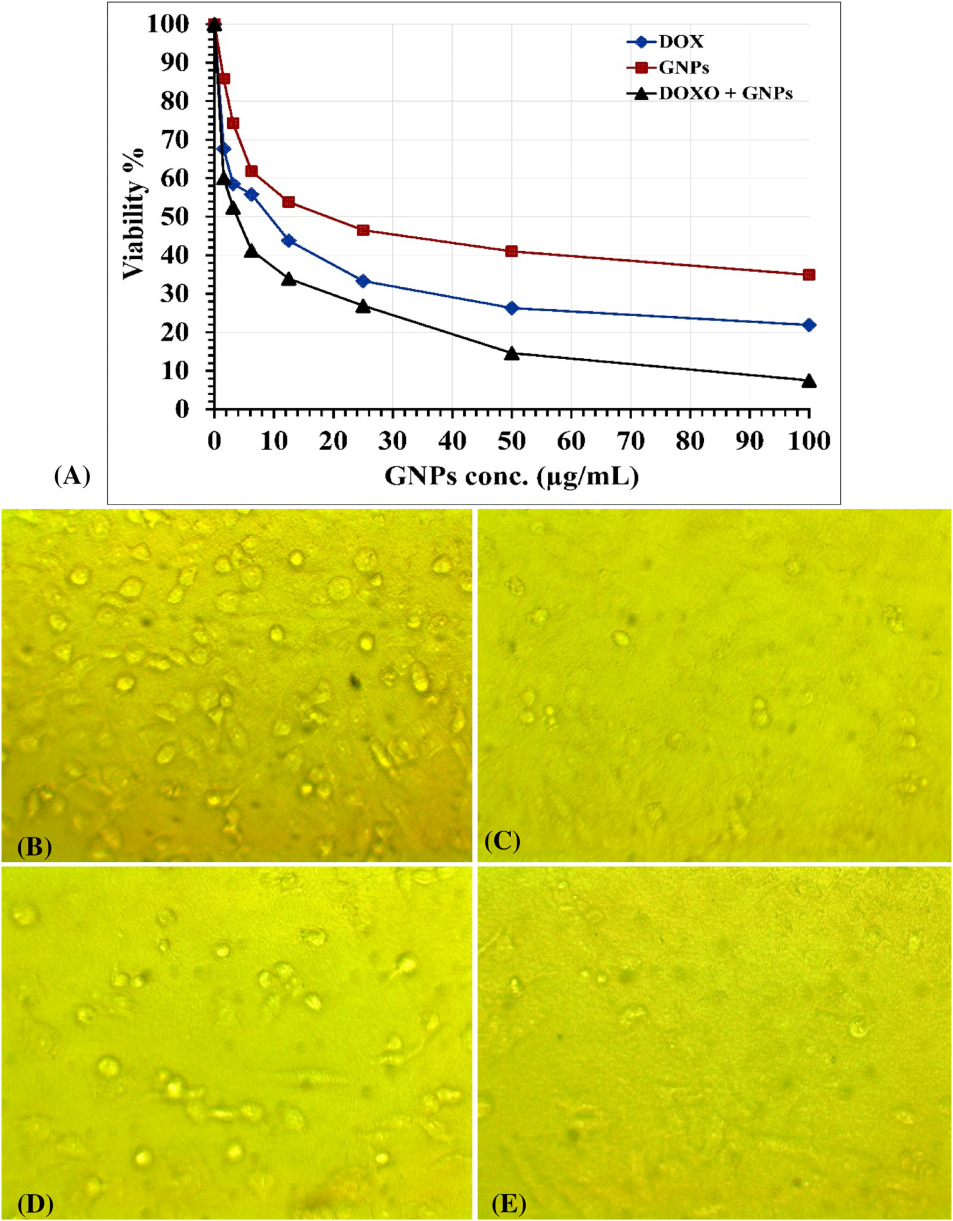
(**A**) In vitro concentration—response plots of HeP-G2 cell line against treatment with Dox (positive control), GNPs and a combination of Dox and GNPs. (**B**, **C**, **D**, **E**) images of HeP-G2 cell line (control), treated with Dox (positive control), GNPs and a combination of Dox and GNPs. These images are taken with inverted microscope lenses ×10.

The MTT assay was employed to assess the anticancer effectiveness of GNPs synthesized by *S. albogriseolus* in comparison to doxorubicin (Dox) against the HeP-G2 human cancer cell line in vitro. The results obtained indicated that GNPs displayed cytotoxic properties against the tested cell line shown in Fig. 9A–E. Dox significantly reduced HeP-G2 cell viability, exhibiting an IC_50_ value of 7.26 ± 0.4 µg/mL. Moreover, GNPs-based treatments highlighted their potential to destroy cancer cells, with an IC_50_ value of 22.13 ± 1.3 µg/mL. Interestingly, treatments combining Dox and GNPs together showed an IC_50_ value of 3.52 ± 0.1 µg/mL, indicating that they were more effective in killing cancer cells.

In the study of Shanmugasundaram et al.^91^, *Streptomyces nogalater* was used to synthesize GNPs successfully, which were evaluated as anticancer agents against Hep-G2 cells using the MTT assay. The findings of their study indicated significant anticancer activity of the GNPs against Hep-G2 cells after a 24-h treatment, with an observed IC_50_ value of 43.25 µg/mL. The anticancer properties of GNPs that were biologically generated using an extract from *Marsdenia tenacissima* are assessed in the study conducted by Li et al.^92^.

The results of the cytotoxicity test showed that the GNPs were significantly active against cancer cells in terms of cytotoxicity; the IC_50_ value was 59.62 ± 4.37 µg/mL. In a study conducted by Balashanmugam et al.^93^, it was revealed that the IC_50_ of Hep-G2 cells was found to be 30 μg/mL based on in vitro experiments. Nandhini et al.^94^ conducted a study to examine the effects of microbial-mediated GNPs on Hep-G2 cells. They discovered that the IC_50_ value for these GNPs was 10 μg/mL. On the basis of this discovery, more studies were conducted at doses between 10 and 20 μg/mL.

GNPs are becoming increasingly relevant in the detection and treatment of cancer because of their characteristics, including amphiphilicity, shape, biocompatibility, size, carrier capacities, and surface area^95^. According to Yoshioka et al.^96^, many factors, including biological source composition, particle size, surface area, surface chemistry, and surface charge, can affect NPs' biocompatibility. During NPs biosynthesis, stabilizing or capping agents are necessary to minimize their toxicity and improve their biocompatibility and bioavailability in living cells, which improves the biomedical activity of the NPs. Also, the capping agents inhibit NPs from being aggregated, improve their ability to remain colloidal, and inhibit the unregulated growth of NPs (particularly those made of metal and metal oxide)^72^. The various forms of capping agents also impact the morphology, size, and catalytic, optical, and magnetic characteristics of the particles.

The cytotoxic impact of GNPs has been attributed to the physicochemical interactions between the atoms of gold and the nitrogen bases and phosphate groups of DNAs, in addition to the functional groups of several intracellular proteins presented in the capping agents^97,98^. Biosynthesized GNPs were thought to have anticancer properties by inducing apoptosis by activating caspase cascades and producing excess ROS, according to Saravanan et al.^99^. The variation of the cytotoxicity responses against Hep-G2 depending on the nature of the source used for GNPs biosynthesis, the morphology, shape, and particle size distribution^100^. The anticancer effect of biosynthesized GNPs against Hep-G2 cells is shape-dependent, according to a study by Lee et al.^101^, who also reported that the nanorods were more cytotoxic than nanostars and nanospheres against Hep-G2 cells. In the study conducted by Ashokkumar et al.^102^, it was discovered that GNPs arrest progression through all stages of the cell cycle, resulting in different levels of DNA content during each phase.

The GNPs were safe for normal cells (HEK-293) and did not have a noticeable effect as there is no clear evidence of nuclear fragmentation upon staining with Hoechst 33344, indicating that the GNPs can be used in a variety of biomedical applications, according to Jeyarani et al.^103^. GNPs with a diameter of 20 nm were less harmful to noncancerous cells in the body and displayed higher therapeutic anticancer activity^104^. No cytotoxicity was found against normal fibroblast cells according to Kajani et al.^105^.

## Conclusion

GNPs have been successfully biosynthesized using the cell-free supernatant of *S. albogriselus* after treating with the HAuCl_4_ aqueous solution. The TEM images showed that the biosynthesized GNPs ranged in size from 5.42 to 13.34. Both CCD and ANN have been applied to analyze, validate, and predict the GNPs biosynthesis using the cell-free supernatant of *S. albogriseolus*. When comparing the prediction capabilities of ANN and CCD, it is shown that ANN has a higher efficiency for accuracy than CCD. Also, the produced NPs showed antitumor potentiality.

## Data Availability

All data generated or analyzed during this study are included in this article.
